# Surveillance of *Trypanosoma cruzi* infection in Triatomine vectors, feral dogs and cats, and wild animals in and around El Paso county, Texas, and New Mexico

**DOI:** 10.1371/journal.pntd.0009147

**Published:** 2021-02-18

**Authors:** Felipe Rodriguez, Brenda S. Luna, Olivia Calderon, Claudia Manriquez-Roman, Karsten Amezcua-Winter, Jonathan Cedillo, Rebeca Garcia-Vazquez, Itzel A. Tejeda, Alvaro Romero, Kenneth Waldrup, Douglas M. Watts, Camilo Khatchikian, Rosa A. Maldonado

**Affiliations:** 1 Department of Biological Sciences, Border Biomedical Research Center, the University of Texas at El Paso, El Paso, Texas, United States of America; 2 New Mexico State University, Department of Entomology, Plant Pathology and Weed Science, Las Cruces, New Mexico, United States of America; 3 Texas Department of State Health Services, Zoonosis Control Region 10, El Paso, Texas, United States of America; Universidad del Valle de Guatemala, GUATEMALA

## Abstract

The causative agent of Chagas disease, *Trypanosoma cruzi*, is transmitted by triatomine vectors. The insect is endemic in the Americas, including the United States, where epidemiological studies are limited, particularly in the Southwestern region. Here, we have determined the prevalence of *T*. *cruzi* in triatomines, feral cats and dogs, and wild animals, the infecting parasite genotypes and the mammalian host bloodmeal sources of the triatomines at four different geographical sites in the U.S.-Mexico border, including El Paso County, Texas, and nearby cities in New Mexico. Using qualitative polymerase chain reaction to detect *T*. *cruzi* infections, we found 66.4% (n = 225) of triatomines, 45.3% (n = 95) of feral dogs, 39.2% (n = 24) of feral cats, and 71.4% (n = 7) of wild animals positive for *T*. *cruzi*. Over 95% of *T*. *cruzi* genotypes or discrete typing units (DTUs) identified were TcI and some TcIV. Furthermore, *Triatoma rubida* was the triatomine species most frequently (98.2%) collected in all samples analyzed. These findings suggest a high prevalence of *T*. *cruzi* infections among triatomines, and feral and wild animals in the studied sites. Therefore, our results underscore the urgent need for implementation of a systematic epidemiological surveillance program for *T*. *cruzi* infections in insect vectors, and feral and wild animals, and Chagas disease in the human population in the southwestern region of the United States.

## Author summary

Chagas disease is caused by the parasite *Trypanosoma cruzi* and one of the major transmission routes is the contaminated feces of blood-feeding triatomine insect vectors, popularly known as kissing bugs. In recent years, this disease has become an important public health concern to the United States and other nonendemic regions of the world. Despite many studies about the prevalence of *T*. *cruzi* in triatomines, and domestic, feral and wild animals in central and southern Texas, there have been no studies in west Texas and New Mexico. In this study, we report the presence of triatomines in residences in El Paso County, TX, and surrounding communities in New Mexico (cities of Anthony and Las Cruces), as well as *T*. *cruzi* infections in feral and wild animals. Using two molecular techniques to analyze the bloodmeal source in triatomines, we detected 12 different mammalian bloodmeal sources, including human and canine. Finally, parasite genotyping showed that most (95%) of the samples belonged to the genotype TcI, which is prevalent in North America. Our findings indicate that the El Paso County and surrounding communities (>950,000 people) are high risk areas for *T*. *cruzi* transmission to humans, feral cats and dogs, and wild animals. Thus, there is an urgent necessity for a public health epidemiological surveillance program for *T*. *cruzi* infections in kissing bugs, feral and wild animals, and in the human population in the U.S.-Mexico border region.

## Introduction

The vector-borne parasite *Trypanosoma cruzi* causes Chagas disease. The parasite infects many mammals including humans, domestic and wild animals in the Americas [1]. An estimated 8 million people are infected in Latin America [2] and approximately 300,000 chronically infected people live in the United States [3]. The infection in humans and animals can be asymptomatic or symptomatic, progressing from an acute phase with flu-like symptoms to a chronic cardiac and/or gastrointestinal (GI) disease that can lead to heart failure, GI megasyndromes and/or sudden death [1,4,5]. There is no human or veterinarian vaccine, and chemotherapy options are of limited efficacy, and exhibit frequent adverse events and variable outcomes [6]. Transmission to mammals occurs after the introduction of infected triatomine fecal material into a wound or mucous membrane, as well as by the oral (consumption of foods and juices contaminated with *T*. *cruzi-*infected kissing bugs or their feces), congenital, and/or transfusion/transplantation routes [7]. The nocturnal triatomine vector, also known as “kissing bug”, serves as the main mode of transmission, particular in established sylvatic and domestic transmission cycles [8]. Around 100 different wildlife mammalian species are competent reservoirs of *T*. *cruzi* [9] and at least 24 species have been recognized as natural wildlife reservoirs in the United States [2], with canines being the most important component of peridomestic transmission, forming a connection between sylvatic and domestic transmission cycles [10,11]. Lastly, human infections can occur when triatomines establish nests near houses and triatomines feed on both humans and animals [12].

There are 141 currently recognized triatomines species in the Americas, many of which can be infected by and transmit *T*. *cruzi* [13]. Of those, 11 species are native to the United States, distributed across the southern half of the country from East to West [2]. Seven of these species have been collected in Texas and all have the potential to transmit *T*. *cruzi* [14]. Although triatomines are an important source of human infections, canines are more likely to be infected with *T*. *cruzi* than humans because of behavioral factors, such as the ingestion of the triatomines by dogs that cause infection through the oral route and because canines commonly sleep outside, increasing the chances of the vector feeding on them [9,15–17]. In Texas, the transmission cycle includes seven reported triatomine species and 27 wild mammalian reservoirs, with many more mammalian reservoirs potentially involved in the cycle [18]. Finally, some human studies in Texas have reported autochthonous transmission of *T*. *cruzi* to humans [19–23].

While there is increasing evidence of Chagas disease in Texas, epidemiologic assessment studies on this disease have not been conducted in southwest Texas, which includes El Paso, an urban border city with a population of over 830,000 [24]. Therefore, in this study, we evaluated the prevalence of *T*. *cruzi* in wild triatomines, and peridomestic stray or feral dogs and cats, and wild animals in El Paso and surrounding communities. All *T*. *cruzi* positive samples were further characterized to determine the genotype or discrete typing units (DTU) of the parasite, an important genetic marker, as well as the mammalian source of the bloodmeals for triatomines. An understanding of the prevalence, distribution, and genetic profile of *T*. *cruzi* in triatomines, and feral and wild animals provided an estimate of the disease risk in both peridomestic and rural settings. Bloodmeal analysis provided critical information on the components of transmission cycles in El Paso area and surrounding areas, as well as transmissibility risks.

## Methods

### Ethics statement

The blood and tissue samples from peridomestic, feral dogs and cats, and wild animals were collected by the veterinarian of the Texas Department of State Health Services, Zoonosis Control Region 10 in the El Paso and surrounding urban and rural communities. The samples from dogs and cats were obtained from a local animal shelter and coordinates of capture location were provided according to the approved protocols A-201408-1, and by the UTEP’s Institutional Animal Care and Use Committee (IACUC) and Institutional Biosafety Committee (IBC) protocol numbers 2014–04, 1608423, and 1111061–1.

### Triatomine collection, identification, and DNA extraction

**Fig 1:**
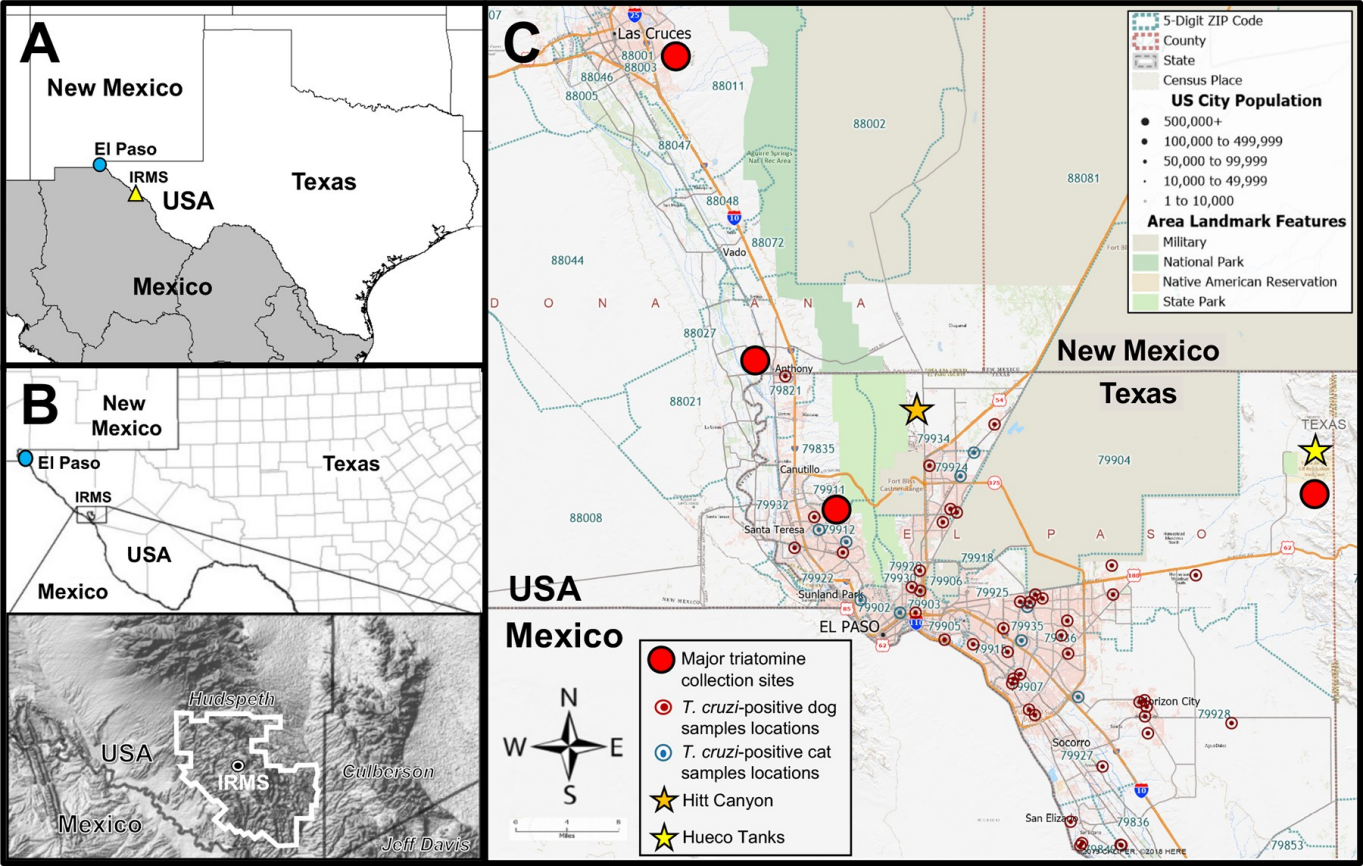
Sample collection sites in El Paso County and surrounding areas in Texas and New Mexico. (**A**) Major triatomine collection sites in or nearby El Paso County (blue circle) and at IRMS, TX (yellow triangle). (**B**) Detailed location and map of IMRS, TX. Inset: IRMS laboratory (encircled black dot) and the field station entire area (white line) are indicated. (**C**) Detailed map of distribution of collected samples in El Paso County, TX, and nearby areas in New Mexico (cities of Anthony and Las Cruces). Maps sourced from the U.S. Geological Survey (https://www.usgs.gov/) and generated with Maptitude 2019 (Caliper Software) and DIVA-GIS 7.5 (https://www.diva-gis.org/).

Triatomine insects were collected from May 2016 to September 2019 (between May and September of each year) in peridomestic and rural areas in El Paso County and Indio Mountains Research Station (IMRS) in Hudspeth, Texas. Collections were also carried out in the urban communities of Anthony and Las Cruces, New Mexico (Fig 1). El Paso is an urban city located in the far southwestern part of Texas bordering Mexico. El Paso city has a population of 681,728, while El Paso County has a population of 839,238 [24,25], in close proximity to two urban communities, the cities of Las Cruces and Anthony, both in Doña Ana County, NM. Las Cruces is located 45 miles northwest of El Paso with a population of 103,432 [26]. Anthony is located 18 miles northwest of El Paso with a population of 9,239 [27]. The other collection site, IMRS, is located 26 miles southwest of Van Horn, TX (121 miles from El Paso). IMRS has no permanent residents, and is used as a field research station by UTEP and as a camping site. Previously, we reported the prevalence of *T*. *cruzi* in IMRS [28]. The triatomine species were identified morphologically [29] and by PCR [30]. Insects were collected by our laboratory members using black light vane trap (BioQuip, Rancho Dominguez, CA) (S1 Fig), by hand collection using tweezers [31,32] (S1 Table), and by members of the communities by unreported *ad hoc* methods (e.g., household items such as cups, pans, plastic bags, etc.). A total of 225 triatomines were captured; 212 were preserved in 95% ethanol, whereas 13 were stored directly at -20°C until analysis. All insects were rinsed in a 1% sodium hypochlorite solution and dissected using sterile instruments in a class-II biosafety cabinet [33]. Dissection was done by carefully removing both pairs of wings, cutting off the connexivum to finally lift up the abdominal wall to reveal the hindgut. The triatomine hindgut was homogenized with a cordless pestle motor (Kimble) in 40 μL phosphate-buffered saline (PBS), pH 7.4. DNA extraction of all samples were done with High Pure PCR Template Preparation Kit (Roche) using the manufacturer’s recommendation. The triatomine genetic material was used to identify the insect species by sequencing a PCR amplification product of mitochondrial DNA, as previously described [30]. Maps were generated with Maptitude 2019 (Caliper Software, Newton, MA) and DIVA-GIS 7.5 (https://www.diva-gis.org/).

### Detection of *T*. *cruzi* in triatomine samples

The samples of triatomine hindguts were tested for *T*. *cruzi* genomic DNA by PCR amplification of a kinetoplastid DNA minicircle, as previously described with minor modifications [34]. Reactions consisted of 200 ng of DNA sample, primers 121 (5’-AAATAATGTACGGG(T/G)GAGATGCATGA-3’) and 122 (5’ GGTCGATTGGGGTTGGTGTAATATA-3’ at final concentration of 0.4 μM each, 12.5 μM 2X GoTaq Green Master Mix (Promega), and nuclease-free water to a final volume of 25 μL. Reactions were run in a 9902 Veriti PCR Thermal Cycler (Applied Biosystems), using the following conditions: incubation at 94°C for 3 min, followed by 35 cycles of 94°C for 30 sec, 57°C for 30 sec, and 72°C for 30 sec. Final extension at 72°C for 7 min and end with at 4°C hold. *T*. *cruzi* (Dm28c clone) genomic DNA positive control was run in all PCR reactions. A positive result was determined by visualization of 330 bp band on a 2% agarose gel. The faint bands were reamplified; the PCR reactions were run again with a double concentration of genomic DNA and the PCR products that showed a very wide band (smear-like) were repeated diluting the amount of DNA to half. The homogeneity in *T*. *cruzi* detection (*i*.*e*., equal proportion of positive outcomes in the PCR reactions) among the 4 locations was tested with a χ^2^ test, setting α = 0.05. The positive ratios in each location were compared using Cohen’s D effect size [35]

### Triatomine bloodmeal analysis

*T*. *cruzi-*positive triatomine samples were analyzed to determine the mammalian species on which they fed. Amplification of the 16S rDNA was accomplished by hemi-nested PCR, as previously described [36] with minor modifications. The first reaction contained 3 μL of DNA template from triatomine, a universal forward primer (VF) (ACC CNT CYM TGT NGC AAA AKR GTG) and a mammalian group-specific forward primer (MF) (CCT GTT TAC CAA AAA CAT CAC) at 0.25 μM final concentration, 12.5 μL 2X GoTaq Green Master mix, and nuclease-free water to a final volume of 25 μL. The secondary reaction contained 2 μL of the primary PCR product, mammalian group-specific reverse primer (MR) (AYT GTC GAT AKG RAC TCT WRA RTA) and MF at 0.5 μM final concentration, 12.5 μL 2X GoTaq Green Master mix, and nuclease-free water to a final volume of 25 μL. A positive triatomine control fed previously with rabbit blood was used in all reactions. Subsequent bi-directional sequencing was run on an ABI 3130x/Genetic analyzer using Bid Dye Terminator chemistries (Applied Biosystems), following the manufacture’s recommendation. Complementary sequences were assembled and verified using CodonCode Aligner V 9.0.1 (CodonCode Corporation, Centerville, MA). Consensus sequences were compared with DNA nucleotide sequences in NCBI BLAST, using the blastn query. Query results with maximum identity equal to or higher than 95% and Expect (E) value equal to or lower than 0 were considered matches. Positive species hits were confirmed to be present in El Paso and surrounding areas based on species distribution using biodiversity databases [37,38]. A total of 41 samples presented mixed template sequencing traces that overlapped, making it impossible to use them. This is because it is known that triatomines may contain DNA from more than one mammalian bloodmeal. To resolve this, restriction enzymes were used for restriction fragment length polymorphism (RFLP) analysis, as described previously [36]. PCR products were singly or doubly digested with Anza 68 *BsuRI* and/or Anza 44 *AluI* (Invitrogen) for 15 min, at 37°C, in a reaction containing 10 U of each enzyme, 1X Anza buffer, 2 μL PCR product and nuclease-free water to a final volume of 20 μL. Enzymes were heat-inactivated at 80°C for 20 min prior to electrophoresis. Products were electrophoresed in a 1.5% agarose gel and visualized with iBright FL1500 imaging system (Thermo Fisher Scientific). Band sizes were estimated using iBright imaging software (Thermo Fisher Scientific) and values compared to controls of the most common mammals found during DNA sequencing (human, rat, mouse, dog, bat, cat, rabbit, horse, and squirrel) and to values previously published [36]. The Shannon-Wiener Index was calculated to compare the bloodmeal diversity at each site, as higher values suggested higher diversity in the site [39, 40], considering only those species confirmed as described above.

### Feral and wild mammal sample collection and DNA extraction

The blood samples from feral or stray dogs and cats were collected from a local animal shelter. Each sample had capture location coordinates determined by GPS (S1 Table). Wild rodents were collected at the Hitt Canyon in the Franklin Mountains State Park in El Paso, TX, using baited Sherman and tomahawk traps. Trapped rodents were identified to species [29], euthanized at the site and the hearts were harvested for analysis. Heart tissues (20–30 mg) were homogenized using gentleMACS M tubes and gentleMACS dissociator (Miltenyi Biotec) in 200 μL PBS. DNA was extracted and each sample was subject to *T*. *cruzi* detection, as described above.

### *T*. *cruzi* DTU determination

All *T*. *cruzi* positive samples were subjected to additional reactions to determine the infecting parasite DTU by multilocus conventional PCR, as previously described [41]. This method was based on the amplification of different *T*. *cruzi* targets. The PCR targets were the following: the intergenic region of Spliced Leader (SL-IRac) to distinguish between TcI (150 bp), TcII, TcV and TcVI (157bp), and TcIII and TcIV (200bp) using primers UTCC (CGTACCAATATAGTACAGAAACTG) and TCac (CTCCCCAGTGTGGCCTGGG). The SL-IR I-II target to differentiate TcI (350 bp) from TcII, TcV and TcVI (300 bp) using primers TCC (CCCCCCTCCCAGGCCACACTG), TC1 (GTGTCCGCCACCTCCTTCGGGCC) and TC2 (CCTGCAGGCACACGTGTGTGTG). The D7 domain of the 24Sα ribosomal DNA target to distinguish between TcII, TcIV and TcVI (140 bp), and TcIII and TcV (125 bp) in a heminested PCR using primers D75 (GCAGATCTTGGTTGGCGTAG) and D76 (GGTTCTCTGTTGCCCCTTTT) (first round) and D76 and D71 (AAGGTGCGTCGACAGTGTGG) (second round). Moreover, the A10 nuclear fragment target to differentiate TcII (580 bp) from TcVI (525 bp) in a heminested PCR using primers Pr1 (CCGCTAAGCAGTTCTGTCCATA) and P6 (GTGATCGCAGGAAACGTGA) (first round) and Pr1 and Pr3M (CGTGGCATGGGGTAATAAAGCA) (second round). The control PCR reactions were performed with 100 pg gDNA extracted from *T*. *cruzi*: Dm28c (TcI), Y (TcII), INPA 3663 (TcIII), CanIII (TcIV), LL014 (TcV), and CL Brener (TcVI). If a sample showed more than one expected band, the presence of more than one infecting parasite DTU was considered and the sample was genotyped as a mix infection.

## Results

### Triatomines collected and *T*. *cruzi* prevalence

A total of 225 triatomines were collected from four different sites, including 21 in El Paso, TX, 33 at IMRS, TX, 139 in Anthony, NM, and 32 in Las Cruces, NM (Fig 1; Table 1). The most common (98.2%, 221/225) species collected was *Triatoma rubida* across all sites, followed by *Triatoma gerstaeckeri* and *Triatoma protracta* (both 0.9%, 2/225), which were both collected in El Paso (Table 1). A total of 150 (66.7%) triatomine samples were positive for *T*. *cruzi* by PCR (Table 1), and of those 147 were *T*. *rubida*, two *T*. *gerstaeckeri*, and one *T*. *protracta*. The prevalence rate of *T*. *cruzi* in triatomines varied along collection locations (χ^2^ = 12.8653, p = 0.002) as follow: Las Cruces, NM (27/32, 84.4%), IMRS (24/33, 72.7%), Anthony, NM (91/139, 65.5%) and El Paso, TX, (5/17, 29.4%) (Table 1). Cohen’s D effect size identified El Paso as having a lower prevalence rate than all other locations (Cohen’s D large effect size >0.5) and Anthony as having a lower prevalence rate than Las Cruces (Cohen’s D medium effect size >0.3) [35].

**Table 1:** *T*. *cruzi-*positive triatomines and DTU genotyping of triatomines collected in the El Paso County, TX, and surrounding rural and urban communities in Texas and New Mexico.

Location	Species	*T*. *cruzi* status P/T ^a^ (%)	DTU			
			TcI	TcIV	TcI+TcIV	ND ^b^
El Paso, TX						
	*T*. *rubida*	5/17 (29.4)	4	0	0	1
	*T*. *protracta* ^c^	1/2 (50.0)	1	0	0	0
	*T*. *gerstaeckeri*^*c*^	2/2 (100.0)	2	0	0	0
IMRS, TX						
	*T*. *rubida*	24/33 (72.7)	22	1	0	1
Anthony, NM						
	*T*. *rubida*	91/139 (65.5)	84	1	5	1
Las Cruces, NM						
	*T*. *rubida*	27/32 (84.4)	25	0	2	0
**Total**		**150/225 (66.7)**	**138**	**2**	**7**	**3**

a P/T, Positive for *T*. *cruzi* per total number of triatomines collected

b ND, Not determined

c Found in Hueco Tanks, near El Paso, TX

### Triatomine bloodmeal analysis

The bloodmeal source was identified in 65.3% (98/150) of the *T*. *cruzi-*positive triatomines collected at three different sites, using two methodologies (DNA sequencing, n = 57, and RFLP, n = 41) (Table 2; S2 Table). Thirteen different mammalian host species were identified. The majority (n = 95; 96.9%) of bloodmeal sources were identified from *T*. *rubida*, one from *T*. *protracta*, and two from *T*. *gerstaeckeri* (Table 2). Furthermore, by bloodmeal DNA sequencing, we found that one of the *T*. *protracta* specimens collected in El Paso, TX, contained a single bloodmeal from *Mus musculus*, whereas by RFLP, we observed that two *T*. *gerstaeckeri* specimens shared a mixed bloodmeal of dog (*Canis* sp.) and mouse (*Mus* sp.). However, unfortunately, we were unable to determine the species of the latter. The greatest blood feeding diversity was detected in Anthony, NM, where 11 different host species served as a source of blood and the value for the Shannon-Weiner Index was 1.67, higher than the values 1.34 and 0.90 found in IMRS and El Paso, respectively. The most frequently host species fed upon by triatomines in El Paso and Anthony was the house mouse, while woodrats were the most frequently preferred host in IMRS. Interestingly, humans were identified as a source of blood for triatomines in Anthony and IMRS (n = 7 and n = 3, respectively). Canid blood was also commonly found in triatomines collected in El Paso and Anthony (n = 4 and n = 10, respectively) (Table 2). The source of blood for 25 triatomines could not be identified by either of the two methods used in this study.

**Table 2:** Identification of mammalian host species that served as a bloodmeal source for *T*. *cruzi-*infected triatomines in El Paso and IMRS, TX, and Anthony, NM, identified by DNA sequencing and RFLP analysis.

Location	DNA sequencing		RFLP^a^	
	Host species	Bloodmeal n	Host species	Bloodmeal n
El Paso, TX	*Mus musculus* (house mouse)	3 ^b^	*Felis* sp. and *Mus* sp.	1
	*Canis lupus familiaris* (domestic dog)	2	*Canis* sp. and *Mus* sp.	2 ^c^
IMRS, TX	*Neotoma leucodon* (white-throated woodrat)	4	*Neotoma* sp. and *Sylvilagus* sp. or *Lepus* sp.	6
	*Homo sapiens* (human)	2	*H*. *sapiens* and *Antrozous* sp.	1
	*Antrozous pallidus* (pallid bat)	2	*Mus* sp. and *Antrozous* sp.	4
			*Mus* sp. and *Sylvilagus* sp. or *Lepus* sp.	4
Anthony, NM	*Mus musculus* (house mouse)	19	*Canis* sp. and *Mus* sp.	4
	*Homo sapiens* (human)	5	*H*. *sapiens* and *Mus* sp.	2
	*Canis lupus familiaris* (domestic dog)	5	*Mus* sp. and *Sylvilagus* sp. or *Lepus* sp.	5
	*Ammospermophilus interpres* (Texas antelope squirrel)	3	*Mus* sp. and *Neotoma* sp.	9
	*Sylvilagus audubonii* (desert cottontail rabbit)	3	*Mus* sp. and *Antrozous* sp.	1
	*Myotis lucifugus* (little brown bat)	3	*Mus* sp. and *Ammospermophilus* sp.	1
	*Lepus californicus* (black-tailed jackrabbit)	2	*Ammospermophilus* sp. and *Sylvilagus* sp. or *Lepus* sp.	1
	*Peromyscus maniculatus* (deer mouse)	1		
	*Perognathus merriami* (Merriam’s pocket mouse)	1		
	*Felis catus* (domestic cat)	1		
	*Equus ferus caballus* (domestic horse)	1		
Total		57		41

a This analysis was done based on the fragment size of the double-digestion with *BsuRI* and *AluI* of the 16S rDNA fragment obtained by PCR, and compared with already known sequences obtained here, and published data [36]. Based on this analysis, most of the digestion fragments had very close molecular sizes, which impaired the identification of the animal species.

b, c Out of the 98 bloodmeals listed in the table, 95 were identified from *T*. *rubida*, one from *T*. *protracta* (^b^), and two from *T*. *gerstaeckeri* (^c^).

### *T*. *cruzi* infection in feral cats and dogs, and wild animals

A total of 95 and 24 blood samples were collected from stray domestic canines and felines, respectively, within the El Paso urban area (Table 3). Among these samples, 45.3% (43/95) of the dogs and 29.2% of the cats (7/24) were positive for *T*. *cruzi* by PCR (Table 3). Additionally, at the Hitt Canyon of the Franklin Mountains State Park, located in El Paso, TX, a total of seven wild animals’ heart samples were collected from two *Chaetodipus intermedius*, and one each of *Peromyscus eremicus*, *Xerospermophilus spilosoma*, *Reithrodontomys megalotis*, *Urocyon cinereoargenteus*, and *Canis latrans*. When analyzed for *T*. *cruzi* infection by PCR, all but two from *C*. *intermedius* (rock pocket mouse) were positive for *T*. *cruzi* (Table 4).

**Table 3:** Prevalence of *T*. *cruzi* infections and DTU genotyping in feral or stray dogs and cats collected in El Paso County, TX.

Species	*T*. *cruzi* status P/T ^a^	DTU			
	(%)	TcI	TcIV	TcI+TcIV	ND ^b^
*Canis lupus* (domestic dog)	43/95 (45.3)	30	9	1	3
*Felis catus* (domestic cat)	7/24 (29.2)	2	1	0	4
Total	50/119 (42.0)	32	10	1	7

a P/T, Positive for *T*. *cruzi* per total number of triatomines collected

b ND, Not determined

**Table 4:** Prevalence of *T*. *cruzi* infection and DTU genotyping in wild animals collected at Hitt Canyon, El Paso County, TX.

Species (Popular name)	n	*T*. *cruzi-*positive	DTU
*Chaetodipus intermedius* (rock pocket mouse)	2	0	NA
*Peromyscus eremicus* (cactus mouse)	1	1	TcI+TcIV
*Xerospermophilus spilosoma* (spotted ground squirrel)	1	1	TcI+TcIV
*Reithrodontomys megalotis* (western harvest mouse)	1	1	TcI
*Urocyon cinereoargenteus* (gray fox)	1	1	TcI
*Canis latrans* (coyote)	1	1	TcI
**Total**	**7**	**5**	

a NA, Not applicable

### *T*. *cruzi* DTUs genotyping

Among all of the *T*. *cruzi*-infected triatomines, and feral and wild animals, the parasite was successfully genotyped in 95.1% of the samples (195/205; Tables 1, 3 and 4). Of the total 150 *T*. *cruzi*-positive triatomines, genotyping revealed that 138 were DTU TcI across all sites, seven from El Paso, 22 from IMRS, 84 from Anthony, and 25 from Las Cruces (Table 1). In addition, two triatomines, one from IMRS and one from Anthony, showed TcIV infection. The genotyping also revealed that seven triatomines had mixed TcI and TcIV (TcI+TcIV) infection (five from Anthony and two from Las Cruces) (Table 1). In the case of feral dogs in El Paso area, TcI was found in 30 animals. TcIV was found in nine canines and only one canine was found to have a mixed TcI+TcIV infection. In felines, TcI was found in two animals and TcIV in one animal (Table 3). Finally, in the wild animals, TcI infection was found in three animals (*R*. *megalotis*, *U*. *cinereoargenteus* and *C*. *latrans*) and two TcI+TcIV mixed infection were found in two animals (*P*. *eremicus and X*. *spilosoma)* (Table 4).

## Discussion

The prevalence of *T*. *cruzi* infection in humans and animals in the U.S. has been recently more studied, since it has become an emerging public health threat in the southeast and southwest regions [9,16,19,22,23,42–47]. In this study, triatomines and blood samples from feral or stray dogs and cats, and wild animals were collected at four different sites in El Paso County and surrounding areas in TX and NM. These samples were tested for *T*. *cruzi* infection, and positive samples were genotyped to determine parasite DTUs. Moreover, triatomine bloodmeal analysis was performed on *T*. *cruzi*-positive samples to identify mammalian host species. This is the first study that determined the prevalence of *T*. *cruzi* infection in feral dogs and cats, wild animals, and triatomine vectors in El Paso County, and surrounding areas in Texas and New Mexico. It is important to highlight that this study also identified the species of regional triatomines, determined the *T*. *cruzi* infection rates in feral and wild animals, genotyped infecting *T*. *cruzi* parasites, and characterized the triatomine bloodmeals, therefore identifying other potential reservoirs of the parasite.

Three different species of triatomines were collected during this study. *T*. *rubida* was the most common species, followed by two species with similar low frequency rate of capture, *T*. *gerstaeckeri* and *T*. *protracta*. These two species with low frequency collection rates were captured at sites in an area named Hueco Tanks, a low rocky mountain rural community located in far east El Paso and worldwide well-known climbing and bouldering site, visited by hundreds of people every year (Fig 1). *T*. *gerstaeckeri* is the most frequently collected species in the U.S., mostly found in southeastern Texas, with a cumulative *T*. *cruzi* infection rate of 57.7%, while *T*. *protracta* has been reported in California, Arizona, New Mexico, and the southern border of Texas with Mexico, with an overall *T*. *cruzi* infection rate of 17.5% [2]. In the case of *T*. *rubida*, this species has been found from western Texas to southern California with a very low cumulative infection rate of 7.2% (much lower rate than what was found in this study) [2]. The present study is the first to report the presence of *T*. *gerstaeckeri* and *T*. *protracta* in El Paso County and surrounding areas. In the case of *T*. *rubida*, a few specimens have been reported since 1949 in El Paso as well as in Ciudad Juarez, a city across the border in Mexico [2,44,48,49]. The only other *Triatoma* sp. reported in El Paso is *Triatoma indictiva*, but no infection status or the exact collection location was available [2]. As for Las Cruces and Anthony, NM, *T*. *protracta* and *T*. *rubida* have been reported, but these reports belong to the Doña Ana County, where these two cities are located, and no exact location has been reported [2]. Here, *T*. *rubida* was also the only species found at IMRS, which is the same species reported by our group in 2015, when 39 *T*. *rubida* specimens were identified [28]. Studies on the biology behavior and spatial distribution of *T*. *rubida*, the most frequently collected species in this study, indicated that this species inhabits environments in close association with their hosts, which includes wild and domestic animals, as well as humans and they are extremely attracted to host-emitted cues, such as body odors, CO_2_, moisture and heat as well as light sources [2,44,50–53]. All of these factors can be found in human residences. Remarkably, all residence locations where triatomines were collected were within the limits of the city of El Paso, next to mountains in semi-rural areas. Furthermore, in El Paso, all residences had domestic dogs and the residents found the triatomines in close proximity to where their dogs slept. It has been reported that dogs are a risk factor for triatomine occurrence in residences forming a transmission bridge for *T*. *cruzi* between human and infected dogs [9,15,16,54]. Thus, a possible explanation for the prevalence of triatomine among residences within city limits of El Paso is that triatomines are attracted to dogs, humans, and light sources. This is also corroborated for triatomines collected from Anthony and Las Cruces, NM, which were collected around lights located in porches and outside buildings. Additionally, studies about the feeding and defecation behavior of *T*. *rubida* indicated this species as an efficient vector [50]. This is because *T*. *rubida* defecates while taking a bloodmeal and it has been proposed that triatomine species that defecate sooner than ten minutes after taking a bloodmeal are effective *T*. *cruzi* transmitter, such as the case for *T*. *rubida* [50, 55–57]. These findings suggest *T*. *rubida* poses a transmission risk of *T*. *cruzi* in El Paso County and surrounding areas, based on distribution, habitat, and feeding behavior.

Of the 225 triatomines collected and analyzed, 150 (66.7%) were positive for *T*. *cruzi*. This infection rate was within the range reported during similar studies conducted in the southern region of the U.S. (range from 41.5 to 80.6% in studies in Texas, Louisiana, and Arizona) [9,11,16,28,43,58–62]. While it is not possible to quantitatively compare infections rates among locations that were sampled using different methodologies, the potential biases resulting from such differences can be inferred to be small enough to allow qualitative comparisons [63]. In this study, Las Cruces, NM, had the highest infection rate (84.4%), which was higher than rates published previously in the southwest, followed by Anthony, NM (65.5%). Interestingly, there has not been any published studies of this nature in New Mexico, even though triatomines have been widely reported in that state [2]. El Paso County had the lowest infection rate of triatomines (8/21, 38.1%) (Table 1). While there is no clear explanation for the lower rate found in El Paso, it is possible that the small number of samples obtained in this location is preventing an accurate estimation of the infection rate. It is possible that the collection of more samples in the future will allow for a more robust estimation of the prevalence rate. Finally, the infection rate at IMRS (72.7%) was higher (15.4% increase) than that reported by our group in the same area in 2015 [28]. These findings demonstrate an active presence of *T*. *cruzi* in triatomines collected in and around El Paso County, TX.

Triatomines are obligate hematophagous and they can feed on any animal, but only mammals are susceptible to *T*. *cruzi* infection [64]. In our study, we used a methodology that allowed for the identification of mammalian host species as the source of triatomine bloodmeals. We only focused on the *T*. *cruzi*-infected insects for the purpose of linking infected triatomines with possible hosts that could have been infected by the insect. Therefore, the mammalian species source of bloodmeals were identified for 98 (79.7%) out of 123 *T*. *cruzi-*positive triatomines collected in residences or in the wild, and immediately immersed in 95% ethanol or frozen until processing. Twenty-seven triatomines from Las Cruces were not taken into consideration for the bloodmeal analysis (Table 2) because they were kept alive in the lab by feeding them rabbit blood. Our first approach detected single bloodmeals in 57 triatomine samples by DNA sequencing. Forty-one triatomine samples resulted in unreadable sequences that contain overlapping base reads (Table 2). This is because it is known that triatomines may contain DNA from more than one mammalian species [11,36,65]. Thus, we opted for a second approach to analyze the DNA by RFLP analysis [36]. By using this method, the mammalian blood source from the 41 aforementioned triatomines, was identified. Finally, failure in the identification of the mammalian bloodmeal source in 25 triatomine samples by both PCR and RFLP analysis could be attributed to: (i) triatomines feeding on non-mammalian hosts such as birds, thus non-amplifiable in the 16S rDNA mammal-specific PCR reaction; or (ii) the lack of a recent bloodmeal, allowing for a degree of blood degradation products inside the insects’ midgut that made the identification by RFLP unfeasible, as previously reported [66–69]. The most diverse source of host bloodmeal were found in Anthony, where triatomine fed on 11 different species of mammals and a higher Shannon-Wiener Index in contrast to El Paso and IMRS, where triatomines fed on 3 and 5 different species, respectively. This may be explained by the fact that Anthony is a semi-rural city with the greatest mammal diversity compared to the other sites. Among the most important results, humans were identified as the source of blood for 7 and 3 triatomines in Anthony and IMRS, respectively, raising the concern for the risk of Chagas disease transmission in these localities, especially at IMRS which is used as a camping and biology field station site. Of all locations, only in El Paso have there been reported cases of *T*. *cruzi-*positive in blood donors, but no cases of autochthonous transmission have been reported [70]. Moreover, domestic dog, domestic cat, and house mouse were found to be a source of blood for triatomines. These animals are important because they live near humans and extensive studies have shown that these animals pose a risk as a source for possible human infection [71,72]. These findings present evidence for potential vector-borne transmission of Chagas disease for humans as well as domestic animals in El Paso County and surrounding areas.

Stray or feral domestic dogs and cats, and wild animals were also tested for *T*. *cruzi*. Domestic dogs are considered as an important reservoir for *T*. *cruzi* in Latin America [17,71,73–81]. It has been proposed that dogs are more likely to acquire *T*. *cruzi* infection due to ingestion of infected triatomines, thus allowing for oral transmission, and that dogs usually sleep outside, increasing therefore the chances of triatomine contact [9,10,17,47,54,82]. Moreover, infection in dogs has been reported from several southern states, including Texas and New Mexico [42,47]. Another animal that lives in close contact with humans are cats. They have been identified as potential sentinels for many infectious diseases because of the environments they share with humans [45]. However, the role of cats in relation to the maintenance and transmissibility of *T*. *cruzi* has not been extensively studied and is not fully understood; the very few studies conducted found a very low prevalence of infection [46,83–86]. In this study, 45.3% (43/95) of feral dogs sampled were positive for *T*. *cruzi*, while 29.2% (7/24) of the feral cats were positive for the parasite (Table 3). Samples from dogs and cats were collected from all over the inner city of El Paso. Furthermore, 71.4% (5/7) of the wild animals tested were positive for *T*. *cruzi*, thus suggesting an important reservoir role. All of the positive species have been previously reported in Texas as *T*. *cruzi* reservoirs [87–90]. Interestingly, triatomine traps were set at sites where *T*. *cruzi*-positive feral dogs and cats, and wild animals were captured, but attempts to collect triatomine at these sites were not successful. This might indicate that the infection in these animals was acquired congenitally. One of the limitations of this part of the study was that data for the age of animals was not available. Such data would have allowed us to analyze the odds of an animal being infected congenitally or through vector transmission, since older animals have a longer opportunity to be exposed to the vector [16,91]. These findings and the substantial number of reported cases (dogs and cats) in Texas and New Mexico discussed in this study may indicate that Chagas disease may occur among feral dogs and cats in the region and that a transmission cycle between vector and animals may have already been established in the western part of Texas.

Finally, the DTUs of all *T*. *cruzi*-positive samples were genotyped with a successful rate of 95.1%. DTUs are important markers for clinical, ecological, and epidemiological features and range from TcI to TcVI and TcBat [92]. Here, TcI was the most predominant genotype, followed by TcIV and some *T*. *cruzi* had a mixed TcI+TcIV infection. Among feral dogs and cats, the equal distribution of DTUs confirmed that there was no difference among them as hosts. TcI is found throughout the Americas with domestic and sylvatic transmission cycles and its associated with chronic Chagas cardiomyopathy [93, 94]. The finding that the most predominant DTU was TcI is an alarming public health concern since chronic Chagas disease is the most challenging form of the disease to be diagnosed and the harder to treat, with possible irreversible hearth damage [95–97]. On the other hand, TcIV is more involved in sylvatic transmission cycles in northern part of South America and the USA, but its clinical association is not well understood and its risk to humans is unknown [98]. These findings are consistent with the documented DTU characterization of *T*. *cruzi* across Texas and USA, where both DTUs have been reported in humans and an extensive range of mammalian species [2,99].

The rates and the observations that *T*. *cruzi* infection rates ranged from moderate to high level in domestic and wild animals support an active enzootic/endemic transmission cycle in the El Paso and surrounding communities. The observation that the most collected triatomine specimens were *T*. *rubida*, which is considered an efficient vector, and the most observed DTU was TcI, which is causative of the very harmful chronic Chagas cardiomyopathy, indicates a high health risk for these communities that needs to be taken into consideration by the respective health authorities. Additionally, the finding of human, canine and feline bloodmeals in these triatomines suggests the possibility of autochthonous parasite transmission in humans as well as in peridomestic or feral animals in the studied areas. More extensive studies are needed, particularly in the high-populated El Paso County area to better understand the involvement of humans and feral and wild animals in the transmission cycle as well as in the burden of Chagas disease.

## Supporting information

S1 Fig Black light vane trap used in this study to collect triatomines. (PDF)

S1 Table Coordinates of animal capture locations. (XLSX)

S2 Table Triatomine and bloodmeal species identified. (XLSX)

## References

[1] Rassi A Jr, Rassi A, Marin-Neto JA. Chagas disease. Lancet. 2010;375(9723):1388–402. 10.1016/S0140-6736(10)60061-X . 20399979

[2] Bern C, Kjos S, Yabsley MJ, Montgomery SP. *Trypanosoma cruzi* and Chagas’ Disease in the United States. Clin Microbiol Rev. 2011;24(4):655–81. Epub 2011/10/07. 10.1128/CMR.00005-11 21976603 PMC3194829

[3] Manne-Goehler J, Umeh CA, Montgomery SP, Wirtz VJ. Estimating the Burden of Chagas Disease in the United States. PLoS Negl Trop Dis. 2016;10(11):e0005033. Epub 2016/11/08. 10.1371/journal.pntd.0005033 27820837 PMC5098725

[4] Barr SC, Schmidt SP, Brown CC, Klei TR. Pathologic features of dogs inoculated with North American *Trypanosoma cruzi* isolates. Am J Vet Res. 1991;52(12):2033–9. Epub 1991/12/01. . 1789518

[5] Barr SC, Gossett KA, Klei TR. Clinical, clinicopathologic, and parasitologic observations of trypanosomiasis in dogs infected with North American *Trypanosoma cruzi* isolates. Am J Vet Res. 1991;52(6):954–60. Epub 1991/06/01. . 1909105

[6] Urbina JA. Recent clinical trials for the etiological treatment of chronic chagas disease: advances, challenges and perspectives. J Eukaryot Microbiol. 2015;62(1):149–56. Epub 2014/10/07. 10.1111/jeu.12184 . 25284065

[7] Coura JR, Dias JC. Epidemiology, control and surveillance of Chagas disease: 100 years after its discovery. Mem Inst Oswaldo Cruz. 2009;104 Suppl 1:31–40. Epub 2009/09/24. 10.1590/s0074-02762009000900006 . 19753455

[8] Lazzari CR, Pereira MH, Lorenzo MG. Behavioural biology of Chagas disease vectors. Mem Inst Oswaldo Cruz. 2013;108 Suppl 1:34–47. Epub 2014/01/30. 10.1590/0074-0276130409 24473801 PMC4109178

[9] Garcia MN, O’Day S, Fisher-Hoch S, Gorchakov R, Patino R, Feria Arroyo TP, et al. One Health Interactions of Chagas Disease Vectors, Canid Hosts, and Human Residents along the Texas-Mexico Border. PLoS Negl Trop Dis. 2016;10(11):e0005074. Epub 2016/11/11. 10.1371/journal.pntd.0005074 27832063 PMC5104435

[10] Beard CB, Pye G, Steurer FJ, Rodriguez R, Campman R, Peterson AT, et al. Chagas disease in a domestic transmission cycle, southern Texas, USA. Emerg Infect Dis. 2003;9(1):103–5. Epub 2003/01/21. 10.3201/eid0901.020217 12533289 PMC2873735

[11] Kjos SA, Marcet PL, Yabsley MJ, Kitron U, Snowden KF, Logan KS, et al. Identification of bloodmeal sources and *Trypanosoma cruzi* infection in triatomine bugs (Hemiptera: Reduviidae) from residential settings in Texas, the United States. J Med Entomol. 2013;50(5):1126–39. Epub 2013/11/05. 10.1603/me12242 24180119 PMC3932564

[12] Wozniak EJ, Lawrence G, Gorchakov R, Alamgir H, Dotson E, Sissel B, et al. The Biology of the Triatomine Bugs Native to South Central Texas and Assessment of the Risk They Pose for Autochthonous Chagas Disease Exposure. J Parasitol. 2015;101(5):520–8. Epub 2015/07/15. 10.1645/15-748 . 26168214

[13] Schofield CJ, Galvao C. Classification, evolution, and species groups within the Triatominae. Acta Trop. 2009;110(2–3):88–100. Epub 2009/04/23. 10.1016/j.actatropica.2009.01.010 . 19385053

[14] Kjos SA, Snowden KF, Olson JK. Biogeography and *Trypanosoma cruzi i*nfection prevalence of Chagas disease vectors in Texas, USA. Vector Borne Zoonotic Dis. 2009;9(1):41–50. Epub 2008/09/20. 10.1089/vbz.2008.0026 . 18800865

[15] Curtis-Robles R, Zecca IB, Roman-Cruz V, Carbajal ES, Auckland LD, Flores I, et al. *Trypanosoma cruzi* (Agent of Chagas Disease) in Sympatric Human and Dog Populations in "Colonias" of the Lower Rio Grande Valley of Texas. Am J Trop Med Hyg. 2017;96(4):805–14. Epub 2017/02/09. 10.4269/ajtmh.16-0789 28167589 PMC5392625

[16] Curtis-Robles R, Snowden KF, Dominguez B, Dinges L, Rodgers S, Mays G, et al. Epidemiology and Molecular Typing of *Trypanosoma cruzi* in Naturally-Infected Hound Dogs and Associated Triatomine Vectors in Texas, USA. PLoS Negl Trop Dis. 2017;11(1):e0005298. Epub 2017/01/18. 10.1371/journal.pntd.0005298 28095511 PMC5287457

[17] Tenney TD, Curtis-Robles R, Snowden KF, Hamer SA. Shelter dogs as sentinels for *Trypanosoma cruzi* transmission across Texas. Emerg Infect Dis. 2014;20(8):1323–6. Epub 2014/07/26. 10.3201/eid2008.131843 25062281 PMC4111162

[18] Gunter SM, Brown EL, Gorchakov R, Murray KO, Garcia MN. Sylvatic Transmission of *Trypanosoma cruzi* Among Domestic and Wildlife Reservoirs in Texas, USA: A Review of the Historical Literature. Zoonoses Public Health. 2017;64(5):313–27. Epub 2016/12/03. 10.1111/zph.12330 . 27911051

[19] Garcia MN, Hotez PJ, Murray KO. Potential novel risk factors for autochthonous and sylvatic transmission of human Chagas disease in the United States. Parasit Vectors. 2014;7:311. Epub 2014/07/06. 10.1186/1756-3305-7-311 24996479 PMC4094476

[20] Garcia MN, Burroughs H, Gorchakov R, Gunter SM, Dumonteil E, Murray KO, et al. Molecular identification and genotyping of *Trypanosoma cruzi* DNA in autochthonous Chagas disease patients from Texas, USA. Infect Genet Evol. 2017;49:151–6. Epub 2017/01/18. 10.1016/j.meegid.2017.01.016 . 28095298

[21] Gunter SM, Murray KO, Gorchakov R, Beddard R, Rossmann SN, Montgomery SP, et al. Likely Autochthonous Transmission of *Trypanosoma cruzi* to Humans, South Central Texas, USA. Emerg Infect Dis. 2017;23(3):500–3. Epub 2017/02/22. 10.3201/eid2303.161157 28221110 PMC5382766

[22] Garcia MN, Aguilar D, Gorchakov R, Rossmann SN, Montgomery SP, Rivera H, et al. Evidence of autochthonous Chagas disease in southeastern Texas. Am J Trop Med Hyg. 2015;92(2):325–30. Epub 2014/11/06. 10.4269/ajtmh.14-0238 25371187 PMC4347336

[23] Harris N, Woc-Colburn L, Gunter SM, Gorchakov R, Murray KO, Rossmann S, et al. Autochthonous Chagas disease in the southern United States: A case report of suspected residential and military exposures. Zoonoses Public Health. 2017;64(6):491–3. Epub 2017/04/19. 10.1111/zph.12360 . 28418113

[24] United States Census Bureau. QuickFacts: El Paso County, Texas U.S.A.: U.S. Census Bureau; 2019 [cited 2020 October 2, 2020]. Available from: https://www.census.gov/quickfacts/fact/table/elpasocountytexas/PST045219.

[25] Census Bureau U.S. QuickFacts: El Paso city, Texas U.S.A.: U.S. Census Bureau; 2019 [cited 2020 October 2, 2020]. Available from: https://www.census.gov/quickfacts/fact/table/elpasocitytexas/PST045219.

[26] U.S. Census Bureau. QuickFacts: Las Cruces city, New Mexico U.S.A.: U.S. Census Bureau; 2019 [cited 2020 October 2, 2020]. Available from: https://www.census.gov/quickfacts/fact/table/lascrucescitynewmexico/PST045219.

[27] U.S. Census Bureau. QuickFacts: Anthony city, New Mexico U.S.A.: U.S. Census Bureau; 2019 [cited 2020 October 2, 2020]. Available from: https://www.census.gov/quickfacts/fact/table/anthonycitynewmexico/PST045219.

[28] Buhaya MH, Galvan S, Maldonado RA. Incidence of *Trypanosoma cruzi* infection in triatomines collected at Indio Mountains Research Station. Acta Trop. 2015;150:97–9. Epub 2015/07/21. 10.1016/j.actatropica.2015.07.004 26193424 PMC4659426

[29] Tekiela S. Mammals of Texas Field Guide (Mammal Identification Guides). Houston, United States: Adventure Publications, Inc.; 2009 5 28, 2009. 416 p. 10.1007/s00280-008-0816-5

[30] Lyman DF, Monteiro FA, Escalante AA, Cordon-Rosales C, Wesson DM, Dujardin JP, et al. Mitochondrial DNA sequence variation among triatomine vectors of Chagas’ disease. Am J Trop Med Hyg. 1999;60(3):377–86. Epub 1999/08/31. 10.4269/ajtmh.1999.60.377 . 10466963

[31] McGavin G. Insects, spiders, and other terrestrial arthropods. New York, N.Y.: Dorling Kindersley; 2000. 255 p. p.

[32] Drees B. A Guide for Collecting, Preserving and Displaying Insects and Other Arthropods Texas A & M University 1998 [cited 2020 September 14]. Available from: http://bughunter.tamu.edu/

[33] Lent H, Wygodzinsky PW. Revision of the Triatominae (Hemiptera, Reduviidae), and their significance as vectors of Chagas’ disease. New York: American Museum of Natural History; 1979. p. 125–520 p.

[34] Avila H, Goncalves AM, Nehme NS, Morel CM, Simpson L. Schizodeme analysis of *Trypanosoma cruzi* stocks from South and Central America by analysis of PCR-amplified minicircle variable region sequences. Mol Biochem Parasitol. 1990;42(2):175–87. Epub 1990/09/01. 10.1016/0166-6851(90)90160-n . 2270100

[35] Cohen J. Statistical power analysis for the behavioral sciences. 2nd ed. Hillsdale, N.J.: L. Erlbaum Associates; 1988. xxi, 567 p. p.

[36] Roellig DM, Gomez-Puerta LA, Mead DG, Pinto J, Ancca-Juarez J, Calderon M, et al. Hemi-nested PCR and RFLP methodologies for identifying blood meals of the Chagas disease vector, *Triatoma infestans*. PLoS One. 2013;8(9):e74713. Epub 2013/09/17. 10.1371/journal.pone.0074713 24040328 PMC3770599

[37] Schmidly DJ, Bradley RD. The mammals of Texas. Seventh edition (second University of Texas Press edition) ed. Austin: The University of Texas Press; 2016. xxvi, 694 pages p.

[38] Frey J. Taxonomy and distribution of the mammals of New Mexico: an annotated checklist. Occasional Papers, Museum of Texas Tech Univeristy. 2004;240.

[39] Shannon CE. A Mathematical Theory of Communication. Bell Syst Tech J. 1948;27(3):379–423. 10.1002/j.1538-7305.1948.tb01338.x WOS:A1948UH03900001. 30854411

[40] Spellerberg I, Fedor P. A tribute to Claude Shannon (1916–2001) and a plea for more rigorous use of species richness, species diversity and the ‘Shannon–Wiener’ Index. Global Ecology & Biogeography. 2003;12:177–9. 10.1046/j.1466-822X.2003.00015.x

[41] da Cruz Moreira O, Ramirez JC. Genotyping of *Trypanosoma cruzi* from Clinical Samples by Multilocus Conventional PCR. Methods Mol Biol. 2019;1955:227–38. Epub 2019/03/15. 10.1007/978-1-4939-9148-8_17 . 30868531

[42] Bern C, Messenger LA, Whitman JD, Maguire JH. Chagas Disease in the United States: a Public Health Approach. Clin Microbiol Rev. 2019;33(1). Epub 2019/11/30. 10.1128/CMR.00023-19 31776135 PMC6927308

[43] Curtis-Robles R, Auckland LD, Snowden KF, Hamer GL, Hamer SA. Analysis of over 1500 triatomine vectors from across the US, predominantly Texas, for *Trypanosoma cruzi* infection and discrete typing units. Infect Genet Evol. 2018;58:171–80. Epub 2017/12/23. 10.1016/j.meegid.2017.12.016 . 29269323

[44] Curtis-Robles R, Hamer SA, Lane S, Levy MZ, Hamer GL. Bionomics and Spatial Distribution of Triatomine Vectors of *Trypanosoma cruzi* in Texas and Other Southern States, USA. Am J Trop Med Hyg. 2018;98(1):113–21. Epub 2017/11/17. 10.4269/ajtmh.17-0526 29141765 PMC5928729

[45] Esch KJ, Petersen CA. Transmission and epidemiology of zoonotic protozoal diseases of companion animals. Clin Microbiol Rev. 2013;26(1):58–85. Epub 2013/01/09. 10.1128/CMR.00067-12 23297259 PMC3553666

[46] Flores-Ferrer A, Waleckx E, Rascalou G, Dumonteil E, Gourbiere S. *Trypanosoma cruzi* transmission dynamics in a synanthropic and domesticated host community. PLoS Negl Trop Dis. 2019;13(12):e0007902. Epub 2019/12/14. 10.1371/journal.pntd.0007902 . 31834879 PMC6934322

[47] Meyers AC, Meinders M, Hamer SA. Widespread *Trypanosoma cruzi* infection in government working dogs along the Texas-Mexico border: Discordant serology, parasite genotyping and associated vectors. PLoS Negl Trop Dis. 2017;11(8):e0005819. Epub 2017/08/09. 10.1371/journal.pntd.0005819 28787451 PMC5560752

[48] Sullivan TD, McGregor T, Eads RB, Davis DJ. Incidence of *Trypanosoma cruzi*, Chagas, in Triatoma Hemiptera, Reduviidae) in Texas. Am J Trop Med Hyg. 1949;29(4):453–8. Epub 1949/07/01. 10.4269/ajtmh.1949.s1-29.453 . 18153045

[49] Ramsey JM, Peterson AT, Carmona-Castro O, Moo-Llanes DA, Nakazawa Y, Butrick M, et al. Atlas of Mexican Triatominae (Reduviidae: Hemiptera) and vector transmission of Chagas disease. Mem Inst Oswaldo Cruz. 2015;110(3):339–52. Epub 2015/05/21. 10.1590/0074-02760140404 25993505 PMC4489471

[50] Martinez-Ibarra JA, Paredes-Gonzalez E, Licon-Trillo A, Montanez-Valdez OD, Rocha-Chavez G, Nogueda-Torres B. The biology of three Mexican-American species of Triatominae (Hemiptera: Reduviidae): *Triatoma recurva*, *Triatoma protracta* and *Triatoma rubida*. Mem Inst Oswaldo Cruz. 2012;107(5):659–63. Epub 2012/08/02. 10.1590/s0074-02762012000500013 . 22850957

[51] Indacochea A, Gard CC, Hansen IA, Pierce J, Romero A. Short-Range Responses of the Kissing Bug *Triatoma rubida* (Hemiptera: Reduviidae) to Carbon Dioxide, Moisture, and Artificial Light. Insects. 2017;8(3). Epub 2017/08/30. 10.3390/insects8030090 28850059 PMC5620710

[52] Reisenman CE, Gregory T, Guerenstein PG, Hildebrand JG. Feeding and defecation behavior of *Triatoma rubida* (Uhler, 1894) (Hemiptera: Reduviidae) under laboratory conditions, and its potential role as a vector of Chagas disease in Arizona, USA. Am J Trop Med Hyg. 2011;85(4):648–56. Epub 2011/10/07. 10.4269/ajtmh.2011.11-0137 21976567 PMC3183772

[53] Klotz SA, Shirazi FM, Boesen K, Beatty NL, Dorn PL, Smith S, et al. Kissing Bug (*Triatoma* spp.) Intrusion into Homes: Troublesome Bites and Domiciliation. Environ Health Insights. 2016;10:45–9. Epub 2016/04/05. 10.4137/EHI.S32834 27042091 PMC4807888

[54] Kjos SA, Snowden KF, Craig TM, Lewis B, Ronald N, Olson JK. Distribution and characterization of canine Chagas disease in Texas. Vet Parasitol. 2008;152(3–4):249–56. Epub 2008/02/08. 10.1016/j.vetpar.2007.12.021 . 18255233

[55] Zeledon R, Alvarado R, Jiron LF. Observations on the feeding and defecation patterns of three triatomine species (Hemiptera: Reduviidae). Acta Trop. 1977;34(1):65–77. Epub 1977/03/01. . 16468

[56] Martinez-Ibarra JA, Nogueda-Torres B, Montanez-Valdez OD, Michel-Parra JG, Valenzuela-Campos R. Biological Parameters of Two *Triatoma rubida* Subspecies (Hemiptera: Reduviidae) and Their Laboratory Hybrids. J Med Entomol. 2020. Epub 2020/04/21. 10.1093/jme/tjaa069 . 32307539

[57] Martinez-Ibarra JA, Nogueda-Torres B, Gonzalez EP, Alejandre-Aguilar R, Solorio-Cibrian M, Barreto SP, et al. Development of *Triatoma rubida sonoriana*, *Triatoma barberi*, and *Meccus mazzottii* (Heteroptera, Reduviidae) under laboratory conditions. J Am Mosq Control Assoc. 2005;21(3):310–5. Epub 2005/10/29. 10.2987/8756-971X(2005)21[310:DOTRST]2.0.CO;2 . 16252523

[58] Curtis-Robles R, Meyers AC, Auckland LD, Zecca IB, Skiles R, Hamer SA. Parasitic interactions among *Trypanosoma cruzi*, triatomine vectors, domestic animals, and wildlife in Big Bend National Park along the Texas-Mexico border. Acta Trop. 2018;188:225–33. Epub 2018/09/12. 10.1016/j.actatropica.2018.09.002 . 30205082

[59] Gorchakov R, Trosclair LP, Wozniak EJ, Feria PT, Garcia MN, Gunter SM, et al. *Trypanosoma cruzi I*nfection Prevalence and Bloodmeal Analysis in Triatomine Vectors of Chagas Disease From Rural Peridomestic Locations in Texas, 2013–2014. J Med Entomol. 2016;53(4):911–8. Epub 2016/04/24. 10.1093/jme/tjw040 . 27106934

[60] Reisenman CE, Lawrence G, Guerenstein PG, Gregory T, Dotson E, Hildebrand JG. Infection of kissing bugs with *Trypanosoma cruzi*, Tucson, Arizona, USA. Emerg Infect Dis. 2010;16(3):400–5. Epub 2010/03/06. 10.3201/eid1603.090648 20202413 PMC3322010

[61] Moudy RM, Michaels S, Jameson SB, Londono B, Lopez V, Caillouet KA, et al. Factors associated with peridomestic *Triatoma sanguisuga* (Hemiptera: Reduviidae) presence in southeastern Louisiana. J Med Entomol. 2014;51(5):1043–50. Epub 2014/10/04. 10.1603/me13234 . 25276935

[62] Waleckx E, Suarez J, Richards B, Dorn PL. *Triatoma sanguisuga* blood meals and potential for Chagas disease, Louisiana, USA. Emerg Infect Dis. 2014;20(12):2141–3. Epub 2014/11/25. 10.3201/eid2012.131576 25418456 PMC4257814

[63] Ramirez-Sierra MJ, Herrera-Aguilar M, Gourbière S, Dumonteil E. Patterns of house infestation dynamics by non-domiciliated Triatoma dimidiata reveal a spatial gradient of infestation in rural villages and potential insect manipulation by Trypanosoma cruzi. Trop Med Int Health. 2010;15(1):77–86. Epub 2009/11/17. 10.1111/j.1365-3156.2009.02422.x . 19912593

[64] Herman LW, Pedro. Revision of the Triatominae (Hemiptera, Reduviidae), and the significance as vectors of Chagas’ diasease. 1979.

[65] Bosseno MF, Garcia LS, Baunaure F, Gastelum EM, Gutierrez MS, Kasten FL, et al. Identification in triatomine vectors of feeding sources and *Trypanosoma cruzi* variants by heteroduplex assay and a multiplex miniexon polymerase chain reaction. Am J Trop Med Hyg. 2006;74(2):303–5. Epub 2006/02/14. . 16474087

[66] Pizarro JC, Stevens L. A new method for forensic DNA analysis of the blood meal in chagas disease vectors demonstrated using *Triatoma infestans* from Chuquisaca, Bolivia. PLoS One. 2008;3(10):e3585. Epub 2008/11/01. 10.1371/journal.pone.0003585 18974787 PMC2570791

[67] Mota J, Chacon JC, Gutierrez-Cabrera AE, Sanchez-Cordero V, Wirtz RA, Ordonez R, et al. Identification of blood meal source and infection with *Trypanosoma cruzi* of Chagas disease vectors using a multiplex cytochrome b polymerase chain reaction assay. Vector Borne Zoonotic Dis. 2007;7(4):617–27. Epub 2007/11/21. 10.1089/vbz.2007.0106 . 18021027

[68] Gariepy TD, Lindsay R, Ogden N, Gregory TR. Identifying the last supper: utility of the DNA barcode library for bloodmeal identification in ticks. Mol Ecol Resour. 2012;12(4):646–52. Epub 2012/04/05. 10.1111/j.1755-0998.2012.03140.x . 22471892

[69] Mukabana WR, Takken W, Knols BG. Analysis of arthropod bloodmeals using molecular genetic markers. Trends Parasitol. 2002;18(11):505–9. Epub 2002/12/11. 10.1016/s1471-4922(02)02364-4 . 12473367

[70] Garcia MN, Woc-Colburn L, Rossmann SN, Townsend RL, Stramer SL, Bravo M, et al. *Trypanosoma cruzi* screening in Texas blood donors, 2008–2012. Epidemiol Infect. 2016;144(5):1010–3. Epub 2014/08/30. 10.1017/S0950268814002234 . 25170765

[71] Castillo-Neyra R, Chou Chu L, Quispe-Machaca V, Ancca-Juarez J, Malaga Chavez FS, Bastos Mazuelos M, et al. The potential of canine sentinels for reemerging *Trypanosoma cruzi* transmission. Prev Vet Med. 2015;120(3–4):349–56. Epub 2015/05/13. 10.1016/j.prevetmed.2015.04.014 25962956 PMC4657134

[72] Cardinal MV, Sartor PA, Gaspe MS, Enriquez GF, Colaianni I, Gurtler RE. High levels of human infection with *Trypanosoma cruzi* associated with the domestic density of infected vectors and hosts in a rural area of northeastern Argentina. Parasit Vectors. 2018;11(1):492. Epub 2018/09/01. 10.1186/s13071-018-3069-0 30165892 PMC6118006

[73] Minter DM, Minter-Goedbloed E, Marsden PD, Miles MA, Macedo V. Domestic risk factor—an attempt to assess risk of infection with *Trypanosoma cruzi* in houses in Brazil. Trans R Soc Trop Med Hyg. 1973;67(2):290. Epub 1973/01/01. 10.1016/0035-9203(73)90211-3 . 4206114

[74] Mott KE, Mota EA, Sherlock I, Hoff R, Muniz TM, Oliveira TS, et al. *Trypanosoma cruzi* infection in dogs and cats and household seroreactivity to *T*. *cruzi* in a rural community in northeast Brazil. Am J Trop Med Hyg. 1978;27(6):1123–7. Epub 1978/11/01. 10.4269/ajtmh.1978.27.1123 . 103446

[75] Gurtler RE, Cecere MC, Castanera MB, Canale D, Lauricella MA, Chuit R, et al. Probability of infection with *Trypanosoma cruzi* of the vector *Triatoma infestans f*ed on infected humans and dogs in northwest Argentina. Am J Trop Med Hyg. 1996;55(1):24–31. Epub 1996/07/01. . 8702018

[76] Gurtler RE, Cohen JE, Cecere MC, Lauricella MA, Chuit R, Segura EL. Influence of humans and domestic animals on the household prevalence of *Trypanosoma cruzi* in *Triatoma infestans* populations in northwest Argentina. Am J Trop Med Hyg. 1998;58(6):748–58. Epub 1998/07/11. 10.4269/ajtmh.1998.58.748 . 9660458

[77] Estrada-Franco JG, Bhatia V, Diaz-Albiter H, Ochoa-Garcia L, Barbabosa A, Vazquez-Chagoyan JC, et al. Human *Trypanosoma cruzi infection* and seropositivity in dogs, Mexico. Emerg Infect Dis. 2006;12(4):624–30. Epub 2006/05/18. 10.3201/eid1204.050450 16704811 PMC3294681

[78] Barr SC. Canine Chagas’ disease (American trypanosomiasis) in North America. Vet Clin North Am Small Anim Pract. 2009;39(6):1055–64, v-vi. Epub 2009/11/26. 10.1016/j.cvsm.2009.06.004 . 19932362

[79] Pineda V, Saldana A, Monfante I, Santamaria A, Gottdenker NL, Yabsley MJ, et al. Prevalence of trypanosome infections in dogs from Chagas disease endemic regions in Panama, Central America. Vet Parasitol. 2011;178(3–4):360–3. Epub 2011/01/29. 10.1016/j.vetpar.2010.12.043 . 21273002

[80] Montenegro VM, Jimenez M, Dias JC, Zeledon R. Chagas disease in dogs from endemic areas of Costa Rica. Mem Inst Oswaldo Cruz. 2002;97(4):491–4. Epub 2002/07/16. 10.1590/s0074-02762002000400006 . 12118277

[81] Carabarin-Lima A, Gonzalez-Vazquez MC, Rodriguez-Morales O, Baylon-Pacheco L, Rosales-Encina JL, Reyes-Lopez PA, et al. Chagas disease (American trypanosomiasis) in Mexico: an update. Acta Trop. 2013;127(2):126–35. Epub 2013/05/07. 10.1016/j.actatropica.2013.04.007 . 23643518

[82] Gurtler RE, Cardinal MV. Reservoir host competence and the role of domestic and commensal hosts in the transmission of *Trypanosoma cruzi*. Acta Trop. 2015;151:32–50. Epub 2015/06/09. 10.1016/j.actatropica.2015.05.029 . 26051910

[83] Enriquez GF, Cardinal MV, Orozco MM, Schijman AG, Gurtler RE. Detection of *Trypanosoma cruzi* infection in naturally infected dogs and cats using serological, parasitological and molecular methods. Acta Trop. 2013;126(3):211–7. Epub 2013/03/19. 10.1016/j.actatropica.2013.03.001 23499860 PMC3675883

[84] Matos A, Caldart ET, Ferreira FP, Monteiro KC, Souza M, Brunieri D, et al. Antibodies anti-trypanosomatides in domestic cats in Parana: who is at highest risk of infection? Rev Bras Parasitol Vet. 2018;27(2):232–6. Epub 2018/05/31. 10.1590/s1984-296120180033 . 29846450

[85] Jimenez-Coello M, Acosta-Viana KY, Guzman-Marin E, Gomez-Rios A, Ortega-Pacheco A. Epidemiological survey of *Trypanosoma cruzi* infection in domestic owned cats from the tropical southeast of Mexico. Zoonoses Public Health. 2012;59 Suppl 2:102–9. Epub 2013/03/19. 10.1111/j.1863-2378.2012.01463.x . 22958254

[86] Gurtler RE, Cecere MC, Petersen RM, Rubel DN, Schweigmann NJ. Chagas disease in north-west Argentina: association between *Trypanosoma cruzi* parasitaemia in dogs and cats and infection rates in domestic *Triatoma infestans*. Trans R Soc Trop Med Hyg. 1993;87(1):12–5. Epub 1993/01/01. 10.1016/0035-9203(93)90400-k . 8465382

[87] Charles RA, Kjos S, Ellis AE, Barnes JC, Yabsley MJ. Southern plains woodrats (Neotoma micropus) from southern Texas are important reservoirs of two genotypes of *Trypanosoma cruzi* and host of a putative novel *Trypanosoma* species. Vector Borne Zoonotic Dis. 2013;13(1):22–30. Epub 2012/11/07. 10.1089/vbz.2011.0817 23127189 PMC3540927

[88] Kramm MM III, Gutierrez MR, Luepke TD, Soria C, Lopez RR, Cooper SM, et al. *Trypanosoma cruzi* in Free-Ranging Mammalian Populations in South Texas, USA. J Wildl Dis. 2017;53(4):788–94. Epub 2017/05/18. 10.7589/2016-10-232 . 28513328

[89] Aleman A, Guerra T, Maikis TJ, Milholland MT, Castro-Arellano I, Forstner MRJ, et al. The Prevalence of *Trypanosoma cruzi*, the Causal Agent of Chagas Disease, in Texas Rodent Populations. Ecohealth. 2017;14(1):130–43. Epub 2017/01/17. 10.1007/s10393-017-1205-5 . 28091763

[90] Curtis-Robles R, Lewis BC, Hamer SA. High *Trypanosoma cruzi* infection prevalence associated with minimal cardiac pathology among wild carnivores in central Texas. Int J Parasitol Parasites Wildl. 2016;5(2):117–23. Epub 2016/06/23. 10.1016/j.ijppaw.2016.04.001 27330982 PMC4900435

[91] Rowland ME, Maloney J, Cohen S, Yabsley MJ, Huang J, Kranz M, et al. Factors associated with *Trypanosoma cruzi* exposure among domestic canines in Tennessee. J Parasitol. 2010;96(3):547–51. Epub 2010/06/19. 10.1645/GE-2299.1 . 20557201

[92] Zingales B. Trypanosoma cruzi genetic diversity: Something new for something known about Chagas disease manifestations, serodiagnosis and drug sensitivity. Acta Trop. 2018;184:38–52. Epub 2017/09/25. 10.1016/j.actatropica.2017.09.017 . 28941731

[93] Zingales B, Miles MA, Campbell DA, Tibayrenc M, Macedo AM, Teixeira MM, et al. The revised Trypanosoma cruzi subspecific nomenclature: rationale, epidemiological relevance and research applications. Infect Genet Evol. 2012;12(2):240–53. Epub 2012/01/10. 10.1016/j.meegid.2011.12.009 . 22226704

[94] Ramirez JD, Guhl F, Rendon LM, Rosas F, Marin-Neto JA, Morillo CA. Chagas cardiomyopathy manifestations and *Trypanosoma cruzi* genotypes circulating in chronic Chagasic patients. PLoS Negl Trop Dis. 2010;4(11):e899. Epub 2010/12/15. 10.1371/journal.pntd.0000899 21152056 PMC2994916

[95] Bern C, Montgomery SP, Herwaldt BL, Rassi A Jr., Marin-Neto JA, Dantas RO, et al. Evaluation and treatment of chagas disease in the United States: a systematic review. JAMA. 2007;298(18):2171–81. Epub 2007/11/15. 10.1001/jama.298.18.2171 . 18000201

[96] Diez M, Favaloro L, Bertolotti A, Burgos JM, Vigliano C, Lastra MP, et al. Usefulness of PCR strategies for early diagnosis of Chagas’ disease reactivation and treatment follow-up in heart transplantation. Am J Transplant. 2007;7(6):1633–40. Epub 2007/05/22. 10.1111/j.1600-6143.2007.01820.x . 17511688

[97] Afonso AM, Ebell MH, Tarleton RL. A systematic review of high quality diagnostic tests for Chagas disease. PLoS Negl Trop Dis. 2012;6(11):e1881. Epub 2012/11/13. 10.1371/journal.pntd.0001881 23145201 PMC3493394

[98] Miles MA, Llewellyn MS, Lewis MD, Yeo M, Baleela R, Fitzpatrick S, et al. The molecular epidemiology and phylogeography of *Trypanosoma cruzi* and parallel research on Leishmania: looking back and to the future. Parasitology. 2009;136(12):1509–28. Epub 2009/08/21. 10.1017/S0031182009990977 . 19691868

[99] Roellig DM, Savage MY, Fujita AW, Barnabe C, Tibayrenc M, Steurer FJ, et al. Genetic variation and exchange in *Trypanosoma cruzi* isolates from the United States. PLoS One. 2013;8(2):e56198. Epub 2013/03/05. 10.1371/journal.pone.0056198 23457528 PMC3572986

