# Supplementary figures and images for: Surveillance of Trypanosoma cruzi infection in Triatomine vectors, feral dogs and cats, and wild animals in and around El Paso county, Texas, and New Mexico
Source: PLoS Negl Trop Dis. 2021 Feb 18;15(2):e0009147. doi: 10.1371/journal.pntd.0009147 (PMC7924784; doi:10.1371/journal.pntd.0009147)

**Figure S1**

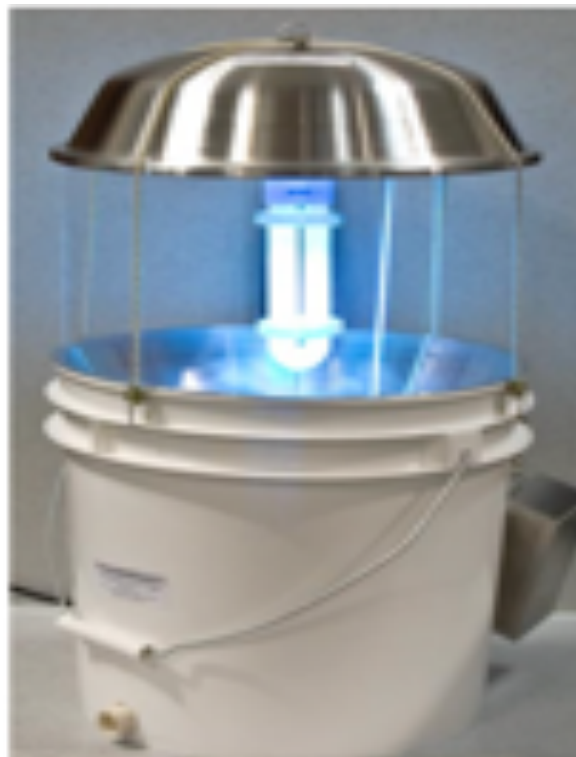

Supplement: S1 Fig — (PDF) [file pntd.0009147.s001.pdf]
